# Supplementary material for: Cell Wall Integrity Pathway Involved in Morphogenesis, Virulence and Antifungal Susceptibility in Cryptococcus neoformans
Source: J Fungi (Basel). 2021 Oct 5;7(10):831. doi: 10.3390/jof7100831 (PMC8540506; doi:10.3390/jof7100831)
Supplement: Supplementary file 1 [file jof-07-00831-s001.zip › jof-1385047-supplementary.pdf]

Table S1. Components of the CWI pathway in *Saccharomyces cerevisiae* and *Cryptococcus neoformans*

Saccharomyces cerevisiae	*Cryptococcus neoformans*
Cell Surface sensors	
Wsc1	CNAG_03308 homologue Mtl1/Mid2
Wsc2	
Wsc3	
Mid2	
Mlt1	
GDP/GTP Exchange factors	
Rom1	Rom2
Rom2	Rom20
	Rom21
Protein Kinase C	
Pkc1	Pkc1
GTPases	
Rho1	Rho1
Rho2	Rho10
Rho5	Rho11
MAPK module	
Bck1	Bck1
Mkk1	
Mkk2	Mkk2
Slt2	Mpk1
Transcription factors	
Rlm1	CNAG_03998 (homologue Rlm1)
Swi4	CNAG_07464 (homologue Swi4)
Swi6	CNAG_01438 (homologue Swi6)
